# The interrelationship between soul and body: Plato as pioneer of the philosophy of health

**DOI:** 10.1007/s11019-026-10357-z

**Published:** 2026-05-11

**Authors:** Nurdane Şimşek

**Affiliations:** Faculty of Humanities and Social Sciences, Department of Philosophy, Marmara University, Istanbul, Türkiye

**Keywords:** Philosophy of health, Holistic medicine, Psychosomatic interrelationship, Health, Disease

## Abstract

This article argues that Plato was a pioneer of the philosophy of health. It examines the psychosomatic interrelationship between soul (psychē) and body (sōma) primarily in the *Charmides* and the *Timaeus*, with selective references to other dialogues, notably the *Republic* and *Phaedrus*. The central claim is that Plato’s account of the psychosomatic interrelationship can be adequately understood only when its sociopolitical dimension is taken into account. Although this emphasis is relatively novel in contemporary scholarship, it was commonplace in Plato’s milieu to conceive of human beings as members and citizens of the city-state (*polis*). The article further argues that Plato’s approach to psychosomatic phenomena extends beyond states of pain or disease and instead encompasses a holistic vision of health and human flourishing. Accordingly, this study aims to deepen the current understanding of Plato’s philosophy of health. Given that the philosophy of health is an emerging interdisciplinary field, the article also seeks to contribute to its conceptual development.

## Introduction

This article argues that Plato was a pioneer of the philosophy of health. It examines the psychosomatic interrelationship between soul (*psychē*) and body (*sōma*) primarily in the *Charmides* and the *Timaeus*, with selective references to other dialogues, notably the *Republic* and *Phaedrus*. The central claim is that Plato’s account of the psychosomatic interrelationship can be adequately understood only when its sociopolitical dimension is taken into account. Although this emphasis is relatively novel in contemporary scholarship, it was commonplace in Plato’s milieu to conceive of human beings as members and citizens of the city-state (*polis*).

The article further argues that Plato’s approach to psychosomatic phenomena extends beyond states of pain or disease and instead encompasses a holistic vision of health and human flourishing. Accordingly, this study aims to deepen the current understanding of Plato’s philosophy of health. Given that the philosophy of health is an emerging interdisciplinary field, the article also seeks to contribute to its conceptual development.

In recent decades, scholarly attention to the body has increased across diverse disciplines, including philosophy, art, psychology, and cultural studies. This growing interest, often referred to as a “renaissance of the body,”^1^ has prompted several related conceptual developments, among which the term “psychosomatic” is particularly significant. In parallel, psychosomatic medicine has evolved rapidly as a distinct medical subdiscipline. This field integrates knowledge from psychology, philosophy, ethics, neuroscience, biology, pharmacology, neurology, internal medicine, and various other medical specialties (Brüne 2015, p. xxi). Despite significant scholarly interest and interdisciplinary study, there is still no consensus on the precise meaning of the term “psychosomatic.”

To address this lack of consensus, the present study first reviews the existing literature on Plato relevant to this topic (II) and then offers a brief historical overview of the term “psychosomatic” and of psychosomatic medicine, aiming to clarify the meaning of the term and the development of the field (III). Section IV introduces Plato’s holistic account of soul and body in the *Charmides* as an essential foundation for understanding the psychosomatic interrelationship. Section V then examines the *Timaeus*, which offers detailed illustrations of this interrelationship.

## Plato and the psychosomatic interrelationship: a critical review of the literature

In his article, “Plato and Psychosomatic Medicine,” physician Nicholas Padis (1952) was among the earliest writers to examine Plato in relation to psychosomatic medicine (pp. 127–129). Padis was writing in an intellectual context shaped by physician Helen Flanders Dunbar (1935), who was instrumental in popularizing the term “psychosomatic” and who, in the “Psyche-Soma” section of *Emotions and Bodily Changes*, placed at its head a Socratic epigraph alluding to Plato’s *Charmides* and emphasizing that the body should not be treated apart from the soul. Although Padis identifies several significant dialogues of Plato as relevant to the subject, his analysis remains brief and preliminary, rather than providing a systematic engagement with the primary texts. Nonetheless, Padis’s contribution remains significant as an early attempt to place Plato within the discourse of psychosomatic medicine and for highlighting a potential problem he termed “psychosomatological determinism.” Padis does not develop this notion in detail, but he uses it to suggest that medicine was entering a new phase in which scientific medicine alone appeared inadequate to the complexity of human life. The present study takes this concern seriously and reconsiders it through a fuller account of the soul–body interrelationship in Plato.

Phillip Sidney Horky (2006), in “The Imprint of the Soul: Psychosomatic Affection in Plato, Gorgias, and the ‘Orphic’ Gold Tablets,” examines psychosomatic affection through the idea that *pathēmata* “mark” or “imprint” the soul (pp. 383–398). He traces this motif across three contexts: Gorgias’ theory of persuasive *logos*, Plato’s eschatological scenes of judgment, and the “Orphic” gold tablets as inscribed ritual media for the soul’s afterlife navigation. His guiding motif is the imprint. Speech, experience, and ritual function as forces that inscribe the soul and render its condition readable. The present article differs from Horky’s study in both scope and aim. Rather than following the imprint metaphor across traditions, it offers a Plato-centered reconstruction of the psychosomatic interrelationship as a framework for health, foregrounding the bidirectional dynamics between soul and body and integrating these dynamics into Plato’s sociopolitical concerns, such as regimen, character formation, and the institutional support of reason in the *polis*.

Hugo Filgueiras de Araújo’s (2023) article, “The psychosomatics attributes in Plato’s *Timaeus* and *Charmides*: disease and health of man,” aligns most closely with the present investigation (pp. 7–22). Filgueiras de Araújo specifically addresses Plato’s *Charmides* and *Timaeus*, alongside the *Republic*, recognizing them as foundational texts for Plato’s psychosomatic understanding. However, his analysis lacks a sustained discussion of the conceptual issues raised by the term “psychosomatic.” An essential aspect of his interpretation is the reciprocal soul–body relationship, leading Filgueiras de Araújo (2023) to conclude that, in Plato, the body should not be devalued and must be considered together with the soul, rather than simply being treated as negligible or wholly subordinate (pp. 8–9). Traditionally, scholars have often characterized Plato’s view of the body negatively, associating it with evil or with being an impediment to truth and virtue. This interpretation is supported by certain descriptions in Plato’s dialogues, such as references to the body as the tomb or prison of the soul.^2^

More recently, however, scholarship has increasingly emphasized Plato’s more positive account of embodiment. A significant contribution in this regard is Coleen P. Zoller’s (2018) book, *Plato and the Body: Reconsidering Socratic Asceticism*. Zoller acknowledges that Plato consistently prioritizes the soul over the body but argues against interpreting Plato’s philosophy as an “austere dualism” (pp. 6–7) marked by the systematic suppression of bodily needs as the definitive marker of philosophical practice.^3^ In line with Zoller’s approach, the present study emphasizes Plato’s positive reflections on embodiment. Although Plato sometimes advocates ascetic ideals, he also recognizes the necessity of engaging with bodily existence, since human life is inevitably embodied. Douglas R. Campbell’s (2022a) formulation of Plato’s characterization of the body as “the soul’s tool” is especially useful here. Thus, while Plato undeniably situates the soul hierarchically above the body, this hierarchy does not imply either a wholesale rejection of the body or its complete disregard.

## The term ‘psychosomatic’ and psychosomatic medicine

The term “psychosomatic” is used in a wide range of contexts, referring to a medical field, clinical approaches, research programs, disorders, and symptoms. Among these usages, “psychosomatic medicine” is the most established; however, Lipowski (1985) notes that the term “medicine,” though fixed by traditional usage, is in one important sense unfortunate, since it connotes a primary concern with disease and thereby suggests an unduly restrictive, disease-centered scope. Psychosomatic medicine, as Lipowski emphasizes, is not merely a set of techniques or a clinical approach but also a scientific discipline, distinguished by its explicit aim to study and formulate explanatory hypotheses concerning the interrelations of biological, psychological, and social phenomena as they pertain to persons. For this reason, Lipowski favors the broader and more neutral term “psychosomatics,” which better captures both the scientific and applied dimensions of the field (pp. 4–5). This terminological clarification provides a useful point of departure for the brief historical sketch that follows.

The origins of psychosomatic inquiry can be traced, in important respects, to ancient Greece. The prefix *psych-* in “psychosomatic” derives from the Greek *psychē* (soul),^4^ and *sōma* translates as “body.” While the modern discipline of psychosomatic medicine is a much later development, Greek medicine, notably Hippocrates, and Greek philosophy, including Plato, already offer influential reflections on the interdependence of bodily condition and psychic life. At the same time, the term “psychosomatic” itself is not found in these ancient texts. The term is often credited to the German physician Johann Christian Heinroth, who in 1818 used it in reference to certain forms of insomnia and to the interaction between soul and body. He also understood soul and body as two aspects of a unified whole, with the body treated as external and the soul as internal (Lipsitt 2006, p. 3).

After its initial introduction, however, the term did not immediately become the basis of a sustained field-wide discourse. It was not until the 1930s that the concept “psychosomatic” began to be consistently explored. The publication of Dunbar’s *Emotions and Bodily Changes: A Survey of Literature on Psychosomatic Interrelationships: 1910–1933* in 1935 marked both the widespread adoption of the term “psychosomatic” and the formal emergence of psychosomatic medicine. Although the field experienced a period of stagnation in the 1950s and 1960s, interest was revived in subsequent decades (Lipowski 1985, p. 122). Nevertheless, the precise meaning of the term “psychosomatic” remains elusive. Dunbar’s title itself points to psychosomatic “interrelationships” rather than to a one-way causal model. However, later clinical practice often privileged mental causes of bodily symptoms, thereby treating the relation as predominantly unidirectional, from mental conflict to bodily distress (Brähler 1988, pp. 10–11).^5^ This shift created a tension between broader historical conceptions of psychosomatic medicine and narrower clinical uses of the term.^6^ A purely unidirectional model obscures the reciprocal interplay between bodily and psychic states that the present study seeks to foreground.

Zbigniew J. Lipowski, a psychiatrist, analyzes the definitions of “psychosomatic” in the *Oxford English Dictionary* to highlight the term’s conceptual ambiguity. As Lipowski notes, the dictionary first included the entry in an appendix published in 1982 (Lipowski 1985, p. 5), offering several definitions. With minor modifications, the current definitions are as follows:

- Involving or depending on both the mind and the body.
- Of, relating to, or designating a physical disorder caused or aggravated by psychological factors.
- Of, relating to, or designating the study of such disorders, or the branch of medicine concerned with this (Oxford English Dictionary, n.d.).

The third entry (2b) defines a discipline rather than the term “psychosomatic” itself. What remains are two working definitions: the first denotes a bidirectional interrelationship between body and mind, while the second identifies a causal path from mind to body. As previously noted, the second definition tends to dominate practical usage. Importantly, in contemporary usage the term “psychosomatic” is not intended to imply the primacy of the mind over the body, nor is it meant to suggest a linear causality. Its structure is conventional rather than hierarchical. In practice, however, “psychosomatic” is frequently interpreted as the mind’s influence on the body. To describe the reverse influence from body to mind, the term “somatopsychic”^7^ has been proposed. Yet this additional term offers little conceptual clarity. First, the term “psychosomatic” already implies a bidirectional interrelationship. Second, the interaction between mind and body is neither linear nor unidirectional but cyclical and shaped by complex, interrelated factors (Lipowski 1985, pp. 5, 7).

This historical asymmetry can be linked in part to the strong influence of psychoanalytic models, which often approached bodily symptoms through their underlying psychic causes and thereby encouraged a predominantly mind-to-body orientation (Brähler 1988, pp. 10–11). It is also reflected in the history of psychosomatic thought, which has often placed greater emphasis on psychosocial factors in the etiology of bodily disease than on psychosocial responses to physical illness or on the influence of somatic processes on psychological functioning (Lipowski 1985, p. 81).^8^

According to Lipowski, the first definition of “psychosomatic” in the *Oxford English Dictionary* carries a philosophical connotation that professionals in the field have often tried to avoid. This definition treats mind and body as separate “entities,” thus evoking a form of dualism, a concept long considered problematic in psychosomatic discourse. Descartes is typically identified as the most influential proponent of dualism, having posited a fundamental division between mind and body that set the stage for debates on their interaction.^9^ Historically, scholars working on psychosomatics have rejected dualism, instead embracing monism, which sees mind and body as unified aspects of the human person. This monistic approach fosters a holistic view of health, in which mental and physical dimensions are seen as interconnected and mutually influential (Lipowski 1985, p. 120). Still, adopting a monist view is not the only way to understand psychosomatic relations. A dualistic framework does not in itself preclude a sophisticated understanding of mind-body interdependence, as Plato himself demonstrates (cf. Sections IV and V).

Over time, the definition of psychosomatic interrelationships has broadened alongside the evolution of conceptual models in the field. It is no longer feasible to imagine mind and body outside their social and cultural context. Humans do not exist in a vacuum:

“*Psychosomatic*” is a term referring or related to the inseparability and interdependence of psychosocial and biologic (physiologic, somatic) aspects of humankind. (This connotation may be called “holistic” as it implies a view of the human being as a whole, a mind-body complex embedded in a social environment.)

“*Psychosomatic medicine*” (psychosomatics) refers to a discipline concerned with (1) the study of the correlations of psychologic and social *phenomena* with physiologic functions, normal or pathologic, and of the interplay of biologic and psychosocial factors in the development, course, and outcome of diseases; and (2) advocacy of a holistic (or biopsychosocial) approach to patient care and application of methods derived from behavioral sciences to the prevention and treatment of human morbidity (Lipowski 1985, p. 133).^10^

This broadened, socially embedded conception of psychosomatic relations provides the conceptual backdrop for understanding Plato’s philosophy of health. Although the social dimension of psychosomatic interrelationships is often regarded as a relatively recent development in contemporary discourse, it was, in fact, a central feature of both Plato’s philosophical outlook and the sociopolitical context of his time. In classical antiquity, the conception of the human being was inseparable from the person’s role as a political agent embedded within the *polis* (Şimşek 2023, pp. 33–35). Section IV turns to Plato’s reconceptualization of the soul and his foundational contribution to a holistic conception of health. Section V then examines the extent to which this social embeddedness informs Plato’s account of psychosomatic relations. It will be argued that, in contrast to many contemporary psychosomatic models that seek to establish a nonhierarchical relationship between soul and body, Plato’s account preserves a distinct ontological asymmetry in which the soul maintains clear primacy over the body.

## *Charmides*: healing the soul, healing the person

In the previous section, the term “psychosomatic” was defined in relation to the concept “holistic.” Both terms are now firmly embedded in the lexicon of contemporary medicine, and in current discourse they are often used interchangeably to denote an integrated view of health and illness. Jan Smuts (1926) coined the term “holism” in his work *Holism and Evolution*. Yet, as with psychosomatics, the conceptual foundations of holism can be traced back to ancient Greece, where its intellectual origins lie in Greek medicine and philosophy. Hippocratic medicine, in particular, has been described as “holistic” since antiquity. Hippocratic holism, whether therapeutic, environmental, or cosmic, treated these domains as integral components of dietetic medicine, capable of complementing one another and, at times, merging into a single explanatory framework (Bartoš 2021, p. 113).

Before turning to the *Charmides*, it is worth asking why this study identifies Plato as the pioneer of the philosophy of health, given that Hippocratic medicine also develops a holistic orientation. Hippocratic “environmental holism” connected a patient’s health to physical factors such as seasons, winds, geography, and habits. These factors were interpreted through the four primary qualities of hot, cold, wet, and dry, which structured humoral diagnosis and guided preventive regimens in coordination with predictable natural changes (Bartoš 2021, pp. 118–122). Plato’s focus, by contrast, lies on the soul and, in relation to it, the *polis*. Building on this point, the present study argues that Plato articulates a philosophically grounded account that connects health not only to the body, but also to the soul and the *polis*.

Early in the dialogue *Charmides*, Socrates’ attention is drawn to the beautiful young man Charmides, and the discussion quickly turns to whether the beauty of his soul matches that of his body. The primary objective of the dialogue is to explore the virtue temperance (*sōphrosunē*). This main topic of the dialogue is inextricably linked to the health of body and soul because “when the soul acquires and possesses temperance, it is easy to provide health both for the head and the rest of the body” (Plato, 1997a, *Charmides*, trans. Sprague, 157a). Charmides’s headache serves as the occasion for the ensuing philosophical exchange. Socrates informs Charmides that he has a remedy in form of a leaf that could relieve Charmides’s pain. Without a charm, however, the leaf would be ineffective.^11^ In explaining the nature of the charm, Socrates first presents a body-centered conception of the whole (*to holon*), which he attributes to the good doctors. According to these practitioners, the treatment of a single part requires the treatment of the whole: to cure an ailment of the eye, one must first heal the head; to heal the head, one must in turn treat the entire body. This process further requires the regulation of diet and regimen through appropriate exercises and practices, thereby addressing both the part and the whole (*Charmides* 156b–156c).

This conception of the whole was already recognized in the Greek world. Socrates acknowledges and endorses this approach in his subsequent reference to a Thracian physician. At the same time, however, he seeks to broaden this holistic understanding beyond the body alone. To this end, he cites the words of the Thracian physician whom he encountered during his military service:

‘But our king Zalmoxis,’ he said, ‘who is a god, says that just as one should not attempt to cure the eyes apart from the head, nor the head apart from the body, so one should not attempt to cure the body apart from the soul. And this, he says, is the very reason why most diseases are beyond the Greek doctors, that they do not pay attention to the whole (*to holon*) as they ought to do, since if the whole is not in good condition, it is impossible that the part should be. Because,’ he said, ‘the soul is the source both of bodily health and bodily disease for the whole man, and these flow from the soul in the same way that the eyes are affected by the head. So it is necessary first and foremost to cure the soul if the parts of the head and of the rest of the body are to be healthy’ (Plato, 1997a, *Charmides*, trans. Sprague, 156d–157a).

At this point, the first question to consider is what Socrates means by “the whole.” In the literature, two primary interpretations are commonly advanced.^12^ According to the first, “the whole” refers to the soul. According to the second, “the whole” designates the soul–body compound, a conception that emphasizes the unity of the person. In the first reading, the soul itself constitutes the whole, whereas in the second it functions as one part of the whole. A third, more nuanced interpretation has been proposed by Korobili and Stefou (2021). They argue that the *Charmides* allows for a flexible usage: in some passages the soul is presented as “the whole,” while in others it is portrayed as part of the soul–body compound.^13^ This approach avoids forcing a single meaning onto every occurrence by showing that Plato’s usage shifts according to context. When the human being is considered as a unified compound, the soul appears as one part of that whole, though it remains the ruling principle. Yet when harmony between body and soul is lost, the soul assumes the role of “the whole” in order to restore balance and reestablish the integrity of the person (Korobili and Stefou 2021, pp. 201–202). For the purposes of the present study, it is sufficient to emphasize that the soul retains priority as the ruling principle, even when the person is understood as a soul–body whole.

So, how should the soul be treated? According to the *Charmides*, the soul is healed through “certain charms, and these charms consist of beautiful words (*kaloi logoi*)” that generate temperance (Plato, 1997a, *Charmides* 157a). Once the soul attains temperance, it becomes easier to achieve health in both soul and body. The *Charmides* is one of Plato’s early dialogues, often classified as Socratic or aporetic, and such dialogues usually conclude without offering a definitive answer. Even so, the dialogue’s lack of a definitive conclusion does not imply that the question of how to heal the soul remains unanswered. Socrates does not directly identify temperance with these “beautiful words.” Rather, he claims that beautiful words generate temperance. From this perspective, the *Charmides* itself may be interpreted as an example of “beautiful words” aimed at fostering temperance, even if it does not provide a definitive definition of temperance. Robinson likewise interprets these “beautiful words” as “noble discourses” or “sound advice” capable of morally improving the person (Robinson 1995, pp. 7–8). Thus, the Socratic method itself can be understood as a form of philosophical therapy that guides individuals toward moral improvement and well-being.

In the literature, Plato’s *Charmides* is often interpreted as presenting philosophy as a form of holistic medical practice, as suggested by Korobili and Stefou’s (2021) article, “Plato’s *Charmides* on Philosophy as Holistic Medical Practice.” A similar approach appears in Edoardo Benati’s (2024) “Zalmoxis’ Medical Holism in the *Charmides*.” However, not all scholars agree that King Zalmoxis’s view should be interpreted as a genuine medical view. Jan Helge Solbakk speaks of a “pseudo-medical conception” and proposes a “transpositional” interpretation, according to which the “medical references” in the *Corpus Platonicum* should not “be considered as purely medical, but as serving a specific function within the structure of the philosophical argument”, namely clarifying philosophical problems (Solbakk 1993, p. 2).^14^ Applied to the *Charmides*, the “transpositional” interpretation claims that the medical references only function as an “‘investigative tool’ in relation to the virtue of σωφροσύνη” (Solbakk 1993, p. 35). To be sure, medical references in Plato’s works often serve the function to clarify philosophical problems and should not always “be considered as purely medical” (Solbakk 1993, p. 2). However, the philosophical issue discussed in the context of King Zalmoxis’s holistic medical views is the virtue of temperance, which leads to health of both body and soul (*Charmides*, 157a). Unlike in contemporary approaches to scientific disciplines, philosophy and medicine were not sharply separated in classical antiquity. For Plato, they were overlapping domains that influenced each other in theory and practice.^15^ As a philosopher of health, Plato maintains that the philosophical problems of virtue and health coincide. In the *Republic* he emphasizes: “Virtue, then, as it seems, would be a certain health, beauty and good condition of a soul” (Plato, 1991, *Republic*, trans. Bloom, 444d–e).^16^ Solbakk’s argument is also problematic because it interprets the early medical references in the *Charmides* based on the aporetic ending of the dialogue.^17^ The next section will discuss whether Plato’s philosophy of health should be characterized as “holistic” and, if so, in what sense.

## *Timaeus*: soul and body as sources of health and disease

The *Charmides* is not the only dialogue in which Plato discusses the notion of “the whole” (*to holon*). Plato addresses this notion again in the considerably later dialogue *Phaedrus*, where he does so through an explicit reference to Hippocrates:

SOCRATES: Do you think, then, that it is possible to reach a serious understanding of the nature of the soul without understanding the nature of the world as a whole?

PHAEDRUS: Well, if we’re to listen to Hippocrates, Asclepius’ descendant, we won’t even understand the body if we don’t follow that method (Plato, 1997b, *Phaedrus*, trans. Nehamas & Woodruff, 270c–d).

As Solbakk notes (1993, p. 107), in the literature there are at least four interpretations of this whole: “one cannot understand the soul without understanding (1) the whole of nature or the universe, (2) the nature of the body as a whole, (3) the nature of the body-soul complex as a whole, or (4) the nature of whatever subject happens to be under discussion as a whole.”^18^ This classification is important because it elucidates that “the whole” is not a fixed concept in Plato’s work, but rather a term that can have different meanings. This is useful for interpreting occurrences in other Platonic dialogues.

As mentioned in the previous section, the *Charmides* begins with the treatment of the body. This brings into view the second possibility of this classification, as it treats human beings as embodied. As the discussion gradually shifts towards the concept of the soul and the ethical question of temperance, the focus shifts to the third possibility.^19^ The fourth possibility is particularly important for this study because it can be adapted to define the relevant whole according to the subjects under discussion. This makes room for the focus of this article: the socio-political conditions that shape psychosomatic interrelations. Within Solbakk’s classification of interpretations of the whole, the *Timaeus* can be read as addressing the first possibility by situating human health within a cosmological order. It further explains how the interrelationship between soul and body is constituted.

Following standard commentaries, the *Timaeus* may be understood as presenting a teleological physiology oriented to ends and education. In what follows, the dialogue is read alongside the *Charmides* in order to develop Plato’s philosophy of health. Cornford traces the dialogue’s progression from cosmology to the seats of the mortal soul and to prescriptions for regimen and exercise (Cornford 1997, pp. 281, 286–287, 349–351). Taylor underscores that the dialogue’s explanatory burden is final-causal, concerned with the purposes of organs and processes rather than with bare material description (Taylor 1928, pp. 629–631). This is why the analysis begins with cosmological and methodological framing before turning to physiological details.

Plato frames his theory of health by a macrocosm–microcosm correspondence: the same cosmological principles that govern the four elements in the cosmos are applied to individual bodies and embodied human beings, so bodily processes are understood through cosmological order (*Timaeus* 82a). This broader framing is consistent with Brisson and Meyerstein’s emphasis on the human being as a microcosm situated within the macrocosm (Brisson and Meyerstein 1995, p. 18). In this way, the “whole” introduced in the *Charmides* is expanded to a cosmic scale. Health and disease are assessed not only within the soul–body compound but also against the ordering principles that structure the cosmos itself. Yet, because this account concerns the sensible, embodied realm, Plato also signals a methodological limit to how exact such explanations can be.

For this reason, Plato characterizes his account of the sensible world as an *eikôs logos*, a “probable account,” rather than an exact *epistēmē* (Plato 1997c, *Timaeus*, trans. Zeyl, 29c–d). As Brisson and Meyerstein (1995) stress, the sensible world is always subject to the disorderly pressures of Necessity (*Anankē*), and for that reason it cannot be grasped with perfect, unassailable knowledge. What is available is a reliable yet inherently tentative explanation (pp. 59–63, 150–180). This methodological modesty is also important for the present argument, because the *Timaeus* can be read alongside the *Republic* and the *Laws* as part of a broader Platonic concern with the conditions under which psychic and bodily order are sustained.

This methodological framing matters for the present argument because it helps explain why Plato does not treat health as a merely technical medical matter confined to individual bodies. Since individual regimen cannot by itself fully master the disorderly pressures of Necessity, the broader political dimension of this problem finds fuller articulation in the *Republic* and the *Laws*, where education and law are presented as supports for rational order in embodied life. In this sense, Plato’s political writings make more explicit a line of thought that remains only partially developed in the *Timaeus*: sociopolitical institutions help reinforce rational order, most systematically through the city-soul analogy in the *Republic* (435e–444a) and, more explicitly, through the appeal to cosmic governance by soul and *nous* as a normative standard for law in *Laws* X (903b–905d). Against this background, Plato’s account of how soul and body are technically connected can now be examined.

To clarify how soul and body are technically connected, Plato begins with the creation of humankind. After crafting the cosmos and the gods, the Demiurge^20^ entrusts the lesser gods with generating mortal creatures: he implants immortal, rational souls, while they fashion mortal bodies and attach the mortal components of the soul, thereby producing human beings. The inevitable outcome is a mortal dimension of the soul characterized by affective states such as fear, pleasure, pain, and boldness (*Timaeus* 41a–d, 69c–d). Accordingly, Plato presents a tripartite psychology: the soul consists of one immortal part and two mortal parts.^21^ The immortal, rational part is located in the head; its separation from the rest of the body by the neck protects it from disturbance by the lower elements. The nobler of the mortal parts, namely the spirited element, is placed in the chest, between the neck and the diaphragm, so that it can ally with and heed the commands of reason, while the appetitive element, the most instinctual aspect of the soul, is assigned to the abdominal region around the liver, below the diaphragm (*Timaeus* 69e–71a). Plato further clarifies how soul and body interrelate by positing a “universal seed,” the marrow, which, together with the spinal column in which it is lodged, links soul and body and “gives root to the human race” (Plato 1997c, *Timaeus*, trans. Zeyl, 73b).^22^ The immortal part resides in the brain, and its connection to the two mortal parts is mediated along the spinal column that extends from the brain, forming an interactive network among the parts of the soul and between soul and body (*Timaeus* 73b–e).

To explore this interrelationship in Plato more deeply, the present study argues that it is crucial to examine health and disease, as well as their underlying causes. Plato distinguishes two kinds of diseases, those of the body and those of the soul. Bodily disease admits of a straightforward account: just as the cosmos is composed of four elements, earth, fire, water, and air, so too is the human body; disease arises when the natural balance among these elements is upset by excess, deficiency, or faulty arrangement (*Timaeus* 82a). The psychosomatic interrelationship becomes explicit when Plato turns to diseases of the soul:

The diseases of the soul that result from a bodily condition come about in the following way. It must be granted, surely, that mindlessness is the disease of the soul, and of mindlessness there are two kinds. One is madness, and the other is ignorance. And so if a man suffers from a condition that brings on either one or the other, that condition must be declared a disease (Plato 1997c, *Timaeus*, trans. Zeyl, 86b–c).

The gravest dangers to the soul arise from excessive pleasures and pains. Overindulgence in pleasure or an intolerant flight from pain clouds perception and can precipitate unreason, even madness. When the body’s marrow “overflows,” like a tree bearing too much fruit, recurrent waves of pleasure and pain tied to desire follow, producing long-lasting disease of the soul. Even so, observers may mistakenly ascribe the result to deliberate vice (*Timaeus* 86b–d). Since Plato introduces this section with the phrase “the diseases of the soul that result from a bodily condition,” one might expect him also to discuss diseases of the soul from non-bodily causes; however, he does not. On this basis, some infer that, for Plato, all diseases of the soul are somatically grounded (Lautner 2011, p. 23; cf. Betegh 2020, p. 253). Campbell (2022b) goes further, arguing that in the *Timaeus* the body is the only cause of psychic disorders.

Plato explains sexual overindulgence (*aphrodisiōn akolasia*) not as an act of willful evil but as a disease of the soul caused by physical conditions in the body (*Timaeus* 86d–87b). For instance, the porousness of the bones allows the seed in the marrow to flow and moisten the body, leading to this condition. Critically, Plato argues that it is wrong to blame people simply as if this condition were chosen in a straightforward sense, because no one is evil by choice; a person becomes bad due to a corrupt bodily condition or a lack of proper education. In a similar way, pain arises from the body and disrupts the soul when acidic or salty phlegm moves without an outlet, mixing with the soul’s movements and causing mental disorders like bad temper, melancholy, or stupidity. Since moral corruption is attributed to factors beyond the individual’s initial control, specifically physiological imbalances and a lack of proper education, the ultimate responsibility and means of therapy necessarily shift to the surrounding social structure (*Timaeus* 87b), thereby reinforcing the central claim of this article that any adequate account of psychosomatic health in Plato must be sociopolitical.

To study the person in isolation from their social context thus reduces and distorts the psychosomatic interrelationship. Plato explicitly links his diagnosis of diseases to the structure of the city-state: moral corruption ensues when people live under deficient governments where harmful rhetoric dominates and corrective education is absent. This suggests that Plato views well-ordered laws and a corrective educational system, rather than individual willpower alone, as the essential framework for reinforcing reason against the inevitability of bodily influence. Taylor’s commentary on *Timaeus* 86b–87b is especially helpful here, since it explicitly treats defective bodily constitution together with bad nurture and connects vice not only with bad physique but also with failures of upbringing and the wider civic environment (Taylor 1928, pp. 343–344). The city–soul model in *Republic* IV makes the point more systematically: disorder in the *polis* corresponds to disorder in the soul, and well-ordered laws and education function as therapy for both (Republic 435e–444a; for the analogy of city and soul, see also Plato’s account in *Republic* VIII and IX of the analogous gradual degeneration of city and soul). In all these instances, Plato represents people as fundamentally embedded within the broader framework of the city-state, and thus explanations and treatments of psychosomatic health and disease must register that framework rather than abstract from it.

The interplay between soul and body is reciprocal: just as the body affects the soul, the soul, in turn, shapes the body. Plato’s reference to the liver makes this vivid. The appetitive part is housed in the lower trunk, at a distance from the reasoning part, and the liver is fashioned smooth and bright, naturally sweet, yet capable of bitterness so that rational thought can be impressed on it “as in a mirror” and reflected as images that move appetite (Plato 1997c, *Timaeus*, trans. Zeyl, 70e–71d). When reason issues threatening thoughts, it mobilizes the bitter element to frighten and discipline appetite, producing bilious discoloration, contraction, and a wrinkled, rough condition; this state is said to result from the bitter response mobilized by reason, not from a mere failure of rational control. By contrast, under gentle guidance reason withholds bitterness and restores the organ to a straight, smooth, and free condition (*Timaeus* 71b–d). When reason is inactive, especially while sleeping, the same imaging arrangement grounds divination in dreams, whose pronouncements require subsequent interpretation by a sound mind (*Timaeus* 71d–72b). On standard interpretations, the liver does not think; it functions as a reflective medium through which rational cognition modulates bodily states and thereby regulates appetite (Lorenz 2012; Moss 2012). Karfík (2012) further locates the appetitive part’s physiological seat in the lower trunk in connection with the liver, aligning the psychology with the teleological physiology.

As discussed in Section II, Padis warns against “psychosomatological determinism,” where psychosomatic diagnoses outpace therapy. Plato’s model avoids this: it allows bidirectional correction through regimen and education, enabling changes in both bodily states and psychic states. A crucial question therefore arises: how can physical diseases originating from the soul and mental diseases stemming from the body be healed? Plato clarifies this issue through an analogy centered on ignorance, which he presents as the gravest disease of the soul. Imagine a body whose legs or another limb are disproportionately large or long. Such a body appears neither beautiful nor functional. Just as a disproportion between the parts of the body is harmful, so is a disproportion between soul and body. A powerful soul can overstimulate the body, resulting in physical exhaustion and disease, particularly during intense intellectual activities or verbal disputes. Conversely, when a large body is joined with a weak soul, physical desires, such as the craving for food, dominate, dulling the soul’s intellectual pursuits and impairing wisdom (*Timaeus* 87e–88b). This analogy is significant because it illustrates how the gravest disease emerges: ignorance results from a disruption in the balance between body and soul. Furthermore, it highlights that the diseases of the soul are not merely intense feelings or desires; rather, the gravest disease of the soul may be located in its inability to perform cognitive functions (Lautner 2011, p. 25).

Building on this analogy between body and soul, proportion emerges as the guiding principle for answering the question of how physical diseases originating from the soul and mental diseases stemming from the body can be healed. For Plato, ignorance results from the disruption of the balance between body and soul and thus from disproportion. In matters of health and disease, virtue and vice, nothing is more essential than the proportion, or disproportion, between soul and body (*Timaeus* 87d). The soul must not grow too strong for the body, nor the body too strong for the soul. The path to health lies in maintaining this delicate equilibrium. Thus, a mathematician or any dedicated intellectual should balance the pursuit of the soul with physical exercise, such as gymnastics. Conversely, those dedicated to physical development should cultivate their souls by applying themselves “to the arts and to every pursuit of wisdom” (Plato 1997c, *Timaeus*, trans. Zeyl, 88c). However, it is crucial to recognize that for Plato this balance is not a matter of equality. As demonstrated in the *Charmides*, the well-being of the soul, which governs the whole, takes precedence over that of the body. Plato maintains the same perspective in the *Timaeus*, emphasizing the importance of prioritizing the care of the soul, the part meant to rule. It is therefore essential to prepare the soul as fully as possible for this task (*Timaeus* 89d).

For the education or treatment of the soul, Plato’s entire corpus, especially his account of the education of the rulers in Book II, III, and VII of the *Republic*, offers a comprehensive example. For the body, the *Timaeus* articulates a hierarchy of therapies: first, self-generated motion guided by understanding^23^, that is, motion arising from within and caused by oneself; second, externally induced motion, for example gentle rocking or sea travel; and, only as a last resort, pharmacological purification (*Timaeus* 88e–89c). Accordingly, Plato prefers regimen and measured exercise as the primary means to purify and restore the body; external rocking can serve when necessary; drugs should be reserved for emergencies, and not for minor ailments, since they often do more harm than good (*Timaeus* 89b–c).^24^ Following Grams, this optimal, internal, reason-guided activity is interpreted here as measured exercise or gymnastics for the sake of clarity, although Plato does not use the term *gymnastikē* at 88e–89c (cf. Plato’s use of *gymnastikē* elsewhere: *Republic* 403e–404e; *Charmides* 156b–156c) (Grams 2009, pp. 186–187). This in turn confirms that the management of bodily regimen is, for Plato, an intrinsically philosophical concern rather than a merely technical medical matter.

## Conclusion

Plato’s philosophy of health, as presented in the *Charmides* and the *Timaeus*, offers a coherent account of the interdependence of soul and body. His emphasis on the necessity of temperance and the cultivation of intellect as the primary means of healing underscores his belief that the well-being of the soul is paramount. Plato recognizes the reciprocal influence between soul and body, acknowledging that physical conditions can affect mental states and vice versa. Consistent with this article’s emphasis on the role of the city-state and its sociopolitical dimensions, this psychosomatic view is nested within a civic framework: education, law, and the *polis* shape, and are shaped by, individual health.

Nevertheless, while Plato recognizes the importance of proportion and balance between soul and body, he does not develop the care of the body as an equally independent and extensive field in its own right. Although the *Timaeus* clearly addresses regimen, exercise, and bodily therapy, these discussions remain integrated into a broader account whose primary emphasis falls on the cultivation and rule of the soul. This relative underdevelopment of bodily care marks a limit of Plato’s account and points to a possible line for further research on how a more fully embodied philosophy of health might be developed from, or in critical dialogue with, his framework. This omission presents an opportunity for further reflection: traditions across periods—ranging from Hellenistic regimens to ancient South and East Asian practices (e.g., yoga and tai chi), as well as their contemporary therapeutic adaptations—could be brought into dialogue with Plato’s framework to extend his vision of health into a more fully embodied account.

## Footnotes

1 For this term, see Brähler (1988), pp. 4–7.

2 See *Phaedo* 82e, *Cratylus* 400c, *Phaedrus* 250c, and *Gorgias* 493a.

3 For a significant recent defense of the body in Plato’s philosophy, see Brown (2025). In “Plato on the Goodness of the Body,” Brown challenges the conventional view that Plato devalues the body, arguing that anti-corporeal readings often overstate the force of ascetic passages and fail to cohere with Plato’s broader metaphysics of goodness. He concludes that, on Plato’s own commitments, the body possesses ontological goodness and is desirable per se.

4 The concept of the soul in ancient Greek thought varies significantly between ordinary Greeks and philosophers such as Plato and Aristotle. For the ordinary Greek, as reflected in Homer’s *Odyssey* (Book 11), the soul is a faint, shadowy entity that descends to the underworld after death, enduring a diminished existence. Achilles famously laments this fate, declaring that he would rather be a servant to a poor farmer on earth than a king among the lifeless dead (lines 488–491). This view contrasts sharply with the philosophical understanding of the soul, particularly in the works of Plato and Aristotle. In the thought of these two philosophers, the soul plays a central role, largely due to the emphasis on its rational faculty. For Plato, the soul is immortal and fundamentally distinct from the body. He divides it into three parts: the rational part, which seeks wisdom and should govern the soul; the spirited part, associated with courage, anger, and ambition; and the appetitive part, driven by bodily desires and material needs (*Republic*, 436a–441c). The soul, according to Plato, is temporarily confined within the body but aspires to attain true knowledge of the eternal Forms, with the Form of the Good as the highest. In the *Phaedrus* (246a–248c), Plato employs the metaphor of a charioteer (representing reason) struggling to guide two horses: one noble and cooperative (spirit) and the other unruly and impulsive (appetite). This allegory illustrates the soul’s internal conflict and its pursuit of harmony and divine understanding. Aristotle, on the other hand, defines the soul as the form of the body, intimately connected to its biological functions and inseparable from it. However, like Plato, he describes the soul in a tripartite structure. The nutritive part, shared with plants, is responsible for growth and reproduction. The sensitive part, common to animals and humans, governs perception and movement. The rational part, unique to humans, enables abstract thought and deliberation (*De Anima*, II.2–3; cf. *Nicomachean Ethics*, I.13). While Aristotle’s conception of the soul is more grounded in biological and functional terms, he nevertheless emphasizes its rational dimension as the defining feature of human nature.

5 Such one-way explanatory models are often discussed under the rubric of psychogenesis. The notion itself has been criticized for implying that psychosomatic medicine treats the psyche as a “morbific agent” and for promoting an overly simple linear chain from emotion to disease (Galdston 1955).

6 This situation reflects Padis’s (1952) warning against “psychosomatological determinism,” as discussed in the previous section.

7 For the use of the term “somatopsychic,” see Arce (1972) and Bransfield and Friedman (2019).

8 Recent work on trauma has also directed attention to body-to-mind processes and to the therapeutic significance of bodily regulation. For a broad account of trauma’s effects on brain, mind, and body, see van der Kolk (2014). For more specific evidence that yoga may reduce PTSD symptoms, see Cramer et al. (2018) and Nejadghaderi et al. (2024).

9 Although Descartes is frequently presented as the paradigmatic defender of mind–body dualism, this characterization requires qualification. While he draws a sharp ontological distinction between mind and body, he also affirms their intimate union and reciprocal interaction: bodily states shape sensation, appetite, and emotion, whereas acts of will produce bodily motion (Descartes 1989, 1996, *Meditation VI*). Accordingly, Descartes’ dualism has been widely criticized, particularly in recent body-centered approaches that dispute any strict separation of mind and body (e.g., Damasio 1994).

10 Lipowski’s emphasis on bidirectionality and on the social dimension of psychosomatic medicine remains a major advance (Lipowski 1985, p. 133). Yet his effort to separate psychosomatic medicine from the philosophical mind-body problem is less persuasive (Lipowski 1985, p. 134). Because psychosomatic inquiry is structured around the relation between mind and body, its central concepts cannot be fully insulated from philosophical assumptions. In this respect, psychosomatic medicine may bracket philosophy for practical purposes, but it cannot entirely escape it.

11 In early civilizations such as Egypt, Babylonia, and Mesopotamia, medical practice was deeply intertwined with religion, magic, and ritual (Longrigg 1963, p. 147). Although Greek medicine retained certain elements of superstition, Greek physicians were the first to pursue rational explanations for illness. Its close association with philosophy enabled medicine to articulate its purpose and methods, gradually shaping it into a systematic body of knowledge (Jaeger 1986, p. 4). Nonetheless, magical practices, including the use of charms and amulets, continued to coexist alongside empirical approaches. In the *Charmides*, Plato presents Socrates as teaching that true bodily health depends upon the health of the soul, with moral discipline serving as a necessary condition for genuine healing (Edelstein 1937, pp. 217, 231).

12 For comprehensive references to the literature, see Korobili and Stefou (2021).

13 See also Solbakk (1993, p. 103).

14 For Solbakk’s understanding of “transposition”, see Solbakk (1993, pp. 2–4).

15 For good reason, Schumacher (1963, p. 237) claims that, for Plato, the true physician must be a philosopher. Medical writers such as Hippocrates, Soranus, and Galen drew on philosophical tools, including causal explanation, systematic classification, and precise conceptual definition, to structure diagnostic and therapeutic approaches. Conversely, philosophers including Plato and Aristotle, as well as Empedocles, pursued empirical investigations into anatomy, physiology, reproduction, and mental illness, thereby enriching medical knowledge. Both fields shared foundational concerns with epistemology, methodology, and practical application (van der Eijk 2005, pp. 8–14; see also Frede 1986, pp. 211–214). It is therefore unsurprising that Plato discusses health and integrates it into his wider philosophical thought.

16 In the *Republic*, Plato defines the virtue temperance (*sōphrosunē*) as “a kind of accord and harmony” (Plato, 1991; trans. Bloom, 430e; see also 431e).

17 Solbakk’s (1993) argument is based on the fifth and sixth definition of temperance in the *Charmides*, which are rather implausible. Although Solbakk admits that the final dialogue with Critias on temperance ends in “a blind alley” (p. 34) and that all six definitions of temperance in the *Charmides* are “finally rejected as not satisfactory” (p. 29, n. 120), he bases his final evaluation of the early medical references in the *Charmides* on the fifth and sixth definition and a remark in *Charmides* 174e2-175a2, which claims that temperance is no prerequisite for health, and that health “belongs to the art of medicine” “conceived as the science of bodily health” (p. 34, 36). However, it is problematic to claim that the discussions around *two* implausible and not satisfactory definitions of temperance, which should not be regarded as “the result of the investigation of σωφροσύνη” (p. 35), refute the extremely plausible earlier interpretation that temperance in the soul leads to both health of the soul and of the body in *Charmides* 157a, which is in line of Plato’s mature view of temperance (*Republic* 444d–e, cf. 430e).

18 Cf. Solbakk’s (1993, pp. 102–106) comprehensive chapter on “The notion of ολον in Corpus Platonicum.”

19 Cf. Solbakk (1993, p. 103).

20 The “Demiurge” (Greek *dēmiourgos*, “craftsman”) in Plato’s *Timaeus* is the benevolent rational cause that orders pre-existent material by looking to the Forms, aiming to produce the best possible cosmos (28a–29b, 29e–30a, 47e–48a). Two interpretations are common in the scholarship: (1) the literal-theological reading, on which the Demiurge is a distinct divine intellect/agent who fashions the world’s order (e.g., Johansen 2004; Sedley 2007); and (2) the symbolic or personification reading, according to which the Demiurge is a heuristic figure in a “likely story” that personifies rational/teleological causation (or the activity of intellect/the Good) rather than a separate deity in Plato’s ontology (e.g., Broadie 2012; Cornford 1997). See also Zeyl’s translation for textual guidance (Plato 1997c).

21 This type of soul has a tripartite structure, which we also find in Plato’s *Republic* (436a–441c).

22 The “universal seed” called the marrow is composed of triangles (Plato 1997c, *Timaeus*, trans. Zeyl, 73b–c). Plato also uses these triangles to explain the phenomenon of death. Over time, the interlocking bonds of the triangles surrounding the marrow weaken and can no longer withstand external pressures. When these bonds break apart, they release the soul’s connections, leading to death (*Timaeus*, 81d).

23 The world-soul’s orderly revolutions both constitute the cosmos’s cognition and maintain the health of its body; human regimen should, as far as possible, imitate this order (*Timaeus* 88c–d; Betegh 2020).

24 Taylor (1928) reads this program as gradual, paideutic treatment rather than abrupt interventions; accordingly, the upshot is a regimen-first medicine that emphasizes self-motion and measured training, while reserving drugs for stubborn cases, in a manner consistent with the Pythagorean emphasis on dietetics and proportion that he traces behind Plato’s practice (pp. 615–619).
